# *Francisella tularensis* subsp. *holarctica* wild-type is able to colonize natural aquatic *ex vivo* biofilms

**DOI:** 10.3389/fmicb.2023.1113412

**Published:** 2023-02-13

**Authors:** Christoph Schaudinn, Kerstin Rydzewski, Beate Meister, Roland Grunow, Klaus Heuner

**Affiliations:** ^1^Centre for Biological Threats and Special Pathogens, Advanced Light and Electron Microscopy (ZBS 4), Robert Koch Institute, Berlin, Germany; ^2^Working Group: Cellular Interactions of Bacterial Pathogens, Centre for Biological Threats and Special Pathogens, Highly Pathogenic Microorganisms (ZBS 2), Robert Koch Institute, Berlin, Germany; ^3^Centre for Biological Threats and Special Pathogens, Highly Pathogenic Microorganisms (ZBS 2), Robert Koch Institute, Berlin, Germany

**Keywords:** *Francisella tularensis*, biofilm, *ex vivo*, survival, microscopy

## Abstract

Biofilms are a matrix-associated lifestyle of microbial communities, often enabling survivability and persistence of such bacteria. The objective of this study was to investigate the survival of the wild-type strain A-271 of *Francisella tularensis* subsp. *holarctica* (*Fth*) in a natural aquatic *ex vivo* biofilm. To that purpose, we allowed *Fth* A-271 to produce its own biofilm on solid surfaces but also to colonize naturally formed biofilms from aquatic habitats, which were infected with *Francisella* in the laboratory. The survival rates of the bacteria in biofilms were compared to those of planktonic bacteria as a function of the employed culture condition. It could be shown by light- and electron microscopy that *Fth* is able to form a complex, matrix-associated biofilm. The biofilm form of *Francisella* showed longer cultivability on agar plates in natural water when compared to planktonic (free-living) bacteria. Be it as a part of the existing *ex vivo* biofilm or free-floating above as planktonic bacteria, more than 80% of *Francisella* were not only able to survive under these conditions for 28 days, but even managed to establish microcolonies and areas with their own exclusive biofilm architecture within the *ex vivo* biofilm. Here, we can demonstrate for the first time that a *Francisella tularensis* wild-type strain (Type B) *is able* to successfully colonize an aquatic multi-species *ex vivo* biofilm. It is worthwhile to speculate that *Fth* might become more persistent in the environment when it forms its own biofilm or integrates in an existing one. Multi-species biofilms have been shown to be more resistant against stress compared to single-species biofilms. This may have an important impact on the long-term survival of *Francisella* in aquatic habitats and infection cycles in nature.

## Introduction

*Francisella tularensis*, the causative agent of tularemia, is found in humans, can produce various clinical symptoms ranging from skin lesions (ulcerous lesion), swollen lymph nodes as well as severe pneumonia, depending on the route of infection. Thus, the disease is defined by the following forms: ulcero-glandular or glandular, oropharyngeal, ocular-glandular and respiratory (Ellis et al., 2002; WHO, 2007; Maurin and Gyuranecz, 2016). *F. tularensis* can infect a wide range of wild animals (Ellis et al., 2002; WHO, 2007). Infections in humans are mostly associated with the highly virulent *F. tularensis* subsp. (*Ft.*) *tularensis* and the less virulent subspecies *Ft. holarctica* (*Fth*) (Keim et al., 2007). However, in individuals with compromised immune systems, opportunistic infections by other *Francisella* species, such as *F. hispaniensis, F. novicida*, *F. salimarina* and *F. philomiragia* have been reported (Hollis et al., 1989; Clarridge et al., 1996; Whipp et al., 2003; Frobose et al., 2020; Hennebique et al., 2022). The family of *Francisellaceae* exhibits also the genus *Allofrancisella*, *Francisella-*like-Endosymbionts of ticks and a new *Francisella* species ([Allo-] *Francisella* sp. strain W12-1067), identified in an aquatic habitat in Germany (Burgdorfer et al., 1973; Rydzewski et al., 2014; Qu et al., 2016; Azagi et al., 2017; Challacombe et al., 2017; Gerhart et al., 2018). So far, it is not known if these species are able to infect humans.

In contrast to *Ftt*, *Fth* is more frequently associated with aquatic habitats and is widely distributed throughout Eurasia (Larson et al., 1955; Oyston et al., 2004; Sjostedt, 2007). *F. tularensis* maintains viability in cold water for long periods of time and it was hypothesized that the aquatic enviroment could serve as a reservoir for *F. tularensis* (Parker et al., 1951; Forsman et al., 2000; Sinclair et al., 2008; Telford and Goethert, 2010; Gilbert and Rose, 2012; Telford and Goethert, 2020). The main habitat of *F. tularensis* in the aquatic reservoir is still unknown and so far it is unclear, if *F. tularensis* is able to multiply within aquatic (bacteria grazing) protozoa (Abd et al., 2003; Thelaus et al., 2009; Buse et al., 2017). However, it is well-known that in (aquatic) natural environments, biofilm formation increases the survival of bacteria. The aquatic habitat-associated species *F. novicida* and *F. philomiragia* are well-known to form biofilms (Durham-Colleran et al., 2010; Margolis et al., 2010; Verhoeven et al., 2010; Van Hoek, 2013; Hennebique et al., 2019; Siebert et al., 2020) and recently it was published that Type A and Type B isolates of *F. tularensis* are also able to form biofilms (Champion et al., 2019; Golovliov et al., 2021; Mlynek et al., 2021). Biofilm formation is influenced by the pH, by phase variation of LPS and capsule (Champion et al., 2019; Mlynek et al., 2021), by stress/ppGpp/relA (Dean et al., 2009; Zogaj et al., 2012), by the two-component system qseC/qseB and BfpR (Durham-Colleran et al., 2010; Dean et al., 2020) and by chitinases and antibiotic susceptibility (Chung et al., 2014; Dean et al., 2015; Biot et al., 2020; Dean et al., 2020). Furthermore, it has been demonstrated that multi-species biofilms are more resistant against stress compared to single-species biofilms (Joshi et al., 2021; Wicaksono et al., 2022) – a property that has not been investigated so far for *F. tularensis*.

Despite of all these attempts, we are still at the beginning to understand the role of *Francisella’s* biofilm formation in its natural environment. We here could demonstrate that a *Fth* wild-type (WT) strain, isolated from a beaver deceased from tularemia, is able to form a matrix-associated biofilm. In addition, we can show for the first time that *Fth* is able to successfully colonize an aquatic multi-species *ex vivo* biofilm.

## Materials and methods

### Strains and growth conditions

Strains used in this study were *Fth* strain LVS (ATCC 29684), *Fth* A-271 WT (Schulze et al., 2016), *Fth* A-271 transfected with pMP814GFP, and *Francisella* sp. strain W12-1067 (Rydzewski et al., 2014).

Plasmid pMP814GFP was generated by infusion cloning. The GroES promoter-Gfp gene construct was introduced from pkk289Km (Broms et al., 2011) into the *Pst*I site of pMP814 (LoVullo et al., 2009) by In-Fusion™ assembly with the Clontech In-Fusion HD Cloning Kit^1^ according to the manufacturer’s instructions.

*Francisella* strains were cultivated in medium T (Pavlovich and Mishan'kin, 1987; Becker et al., 2016), on medium T-based agar plates (MTKH agar: medium T containing 2.4 g l^−1^ of activated charcoal, 14.3 g l^−1^ of agar and 9.5 g l^−1^ of hemoglobin), or on HCA agar (Brain Heart Infusion Agar [Liofilchem, Roseto degli Abruzzi, Italy] with 10% sheep blood). *Fth* A-271 transfected with pMP814GFP was cultivated on *Neisseria* selective medium Plus agar plates (PO5004A, Oxoid, Germany).

*Naegleria gruberi* was cultivated in PYNFH medium (ATCC 1034) at room temperature.The co-culture were performed in an infection buffer (Ac buffer) (for 1 l, 1 g of sodium citrate × 2 H_2_O, 10 ml of 0.4 M MgSO_4_ × 7 H_2_O, 10 ml of 0.25 M Na_2_HPO_4_ × 7 H_2_O, 10 ml of 0.25 M KH_2_PO_4_, 8 ml of 0.05 M CaCl_2_ × 2 H_2_O, 10 ml of 0.005 M Fe(NH_4_)_2_(SO_4_)_2_ × 6 H_2_O.

The mouse or human macrophage-like cell line J774A.1 and U937, respectively, were cultivated in RPMI 1640 + 10% FCS medium (PAA/GE Healthcare Europe GmbH, Freiburg, Germany) at 37°C and 5% CO_2_.

### Survival of *Fth* WT in infection buffer and in co-culture with amoebae

*Naegleria gruberi* was mechanically detached from the flask bottom, transferred to 50 ml tubes and centrifuged at 800× *g* for 10 min. After cell counting in a Neubauer counting chamber (C-Chip, Digital-Bio, DHC-N01), amoebae were adjusted to 5 × 10^5^ cells ml^−1^ in Ac buffer, transferred into wells of a 24-well plate (1 ml/well) and incubated for 2 h at 25°C. Bacteria grown 3 days on MTKH- plates, resuspended in Ac buffer and adjusted to OD_600_ = 1 (corresponding to ~10^9^ CFU ml^−1^). The infection was done with a multiplicity of infection (MOI) of 10 and incubated for up to 28 days at 25°C. In parallel, the same number of bacteria were transferred into the wells of a second 24-well plate containing 1 ml Ac buffer per well, but without amoebae. For colony-forming unit (CFU) determination at various time points, bacteria were harvested by mechanical disruption of the amoebae or directly plated onto agar plates. The agar plates were incubated at 37°C, 5% CO2. Grown bacterial colonies were counted with a colony counter (aCOLyte, Synbiosis, Cambridge, UK) and calculated as bacteria per ml.

### Biofilm formation

#### Biofilm formation at a solid–gas interface

An over-night culture of *Fth* LVS and *Fth* A-271 in medium T was adjusted to OD_600_ = 1. 10 μl (approximately 100 bacteria) were spotted onto each polyester filter (Ø 13 mm, PE02CP01300; Pieper Filter GmbH, Bad Zwischenahn, Germany) located on a MTKH agar plate. This agar plate was incubated at 37°C and 5% CO_2_ for up to 3 days. Every day, one filter was removed and fixed in 4% paraformaldehyde at 4°C for 48 h. For scanning electron microcopy (SEM) visualization, some polyester filters were washed in 50 mM HEPES, dehydrated in 30, 50, 70, 90, 95, 100, 100% ethanol, dried in hexamethyldisilazane, mounted on aluminum stubs, sputter coated with a 12 nm layer of gold–palladium and finally examined in the SEM (ZEISS 1530 Gemini, Carl Zeiss Microscopy GmbH, Germany) operating at 3 kV using the in-lens electron detector. For confocal laser scanning microscopy (CLSM) imaging, some polyester filters were washed in bidest water, stained with DAPI (dsDNA), Sypro® Orange (proteins) and Nile Red (neutral lipids) for 30 min, and imaged with the CLSM (LSM 780, Carl Zeiss Microscopy, Oberkochen, Germany) to visualize biofilm formation at a liquid–solid interface.

In a second set of experiments, the biofilm was generated on Thermanox coverslips (Nunc™ Thermanox™ coverslips, Ø13mm). *Fth* strain A-271 grown on MTKH agar plates at 37°C and 5% CO_2_ for 2 days were used to start a culture (25 ml medium T in a 100 ml flask). After 7 h of incubation at 37°C and 250 rpm, the culture was adjusted to OD_600_ = 0.3. Each well of a 12-well plate containing a Thermanox coverslip was filled with 1 ml of the diluted bacterial suspension and incubated without shaking at 37°C and 5% CO_2_ for 2 days. From day three the plates were shaken at 100 rpm without additional CO_2_ for two weeks, each third day the coverslip was transferred to a new 12-well plate cotaining fresh medium T (1 ml each well). Then the coverslips were transferred into 6-well plates with 2 ml medium per well. During the next 2 weeks, every third day half of the medium T (1 ml) was replaced by fresh medium. This biofilm was then used either for scanning electron microscopy or for the survival experiments in natural water.

### Survival of *Fth* (biofilm and planktonic) in natural water

The Thermanox coverslip with biofilm (see above, 3 days after last replacement with fresh medium T) was removed into a new well and the remaining supernatant was defined as the planktonic bacterial fraction. The coverslip was washed once and filled with 1 ml sterilized natural water (SNW). The supernatant was diluted 1:1000 in SNW. The biofilm as well as the planktonic bacteria were then incubated for a further 28 days at RT (approx. 22.5°C). At day 0, 2, 7, 15, 21 and 28 both bacterial cell types were treated as follows: The biofilm was removed from the coverslip using a cell culture scraper. The whole liquid was then transferred into a 1.5 ml tube and vortexed three times for 45 s. Planctonic cells were also transferred into 1.5 ml tubes and vortexed. Dilutions of all bacterial suspensions were used for viability testing (Live-Dead staining) or plated out onto MTKH agar plates to determine the number of CFU.

In order to gauge the biofilm’s viability, the Thermanox™ coverslips with *Fth* biofilm were stained with the LIVE/DEAD™ BacLight Bacterial Viability kit following the manufacturer’s instructions, and imaged in the CLSM (LSM 780, Carl Zeiss Microscopy, Oberkochen, Germany). Five representative spots of the Thermanox™ coverslips were chosen (center, 3, 6, 9 and 12 o’clock) and images of a standardized area were taken with the 20x NA0.8 objective. The bacteria in the individual green/red channels were counted using ImageJ^2^ (U. S. National Institutes of Health, Bethesda, MD, USA) and recalculated to the area of a Thermanox™ coverslip.

As for the viability of planktonic bacteria, 10 μl of the supernatant were placed in each slot after LIVE/DEAD™ staining of a disposable Neubauer counting chamber (C-Chip™, DigitalBio, NanoEnTek). For safety reasons, the slots were sealed with nail polish (Maybeline, express finish 40 s). Standardized imaging was done using the corner squares (DHC-F01 grid pattern) of each side of the counting chamber. The bacteria in the individual green/red channels were counted using ImageJ (U. S. National Institutes of Health, Bethesda, Maryland, USA, see Footnote 2) and recalculated to one milliliter.

### Natural *ex vivo* biofilm model

In order to obtain natural *ex vivo* biofilms from an environmental aquatic habitat, 5–10 Thermanox™ coverslips were fixed with a distance of approx. 5 cm along a thread. These devices were placed 20–30 cm beneath the water plane of a ditch near Berlin, Germany (Coordinates: 52°, 25 min, 17,84 s north, 13°, 44 min, 58,64 s east) at different seasons (September, November, April and June). The devices were removed after 4 weeks when the coverslips showed a visible biofilm on the surface and transported to the laboratory in a waterfilled bottle. Single coverslips were rinsed with 5 ml SNW from the same water souce wherefrom the biofilm was obtained. The coverslips were placed in wells of a 24-well-plate and covered with 0.2 ml SNW. The cultures were infected with 0.2 ml 10^8^ *Fth* A-271/pMP814GFP or as indicated in SNW on day 0. The cultures were kept at room temperature or at 4°C for the period of time as indicated. The number of colony-forming cells and of genome copies by multiplex real-time PCR targeting *tul4* (*lpnA* gene) were determined after scraping the cells from the coverslips as previously described (Jacob et al., 2019). Suspensions were plated onto selective “Neisseria” agar plates (see strains and growth conditions). GFP-positive clones were counted under UV light and a subset of these bacteria were verified as *Fth* by qPCR analysis. For microscopic analyses, the coverslips were fixed in 4% paraformaldehyde at 4°C for 48 h and processed as described in the method section (biofilm formation).

### Image processing

Images have been cropped and adjusted for optimal brightness and contrast (applied to the entire image) using Adobe Photoshop® (Adobe Systems, San Jose, CA, USA). The image D of Figure 5B was colorized with Adobe Photoshop®.

## Results

### *Francisella* strain A-271 is a virulent wild-type strain of the subspecies *Fth*

#### Intracellular replication and virulence

Here, we investigated the *Fth* wild-type (WT) strain A-271, isolated from the carcass of a beaver deceased from tularemia, and without subsequent serial passaging on agar plates (Schulze et al., 2016). The complete genome sequence of strain A-271 has been determined and displays genes for all classical virulence factors of *Fth* (Sundell et al., 2020). Phylogenetically, this isolate belongs to the B.12 subclade (biovar II, erythromycin resistant) of *Ft. subsp. holarctica*. Strains of biovar II are frequently isolated also from tularemia patients in Germany (Appelt et al., 2019). In addition, we investigated strain A-271 for intracellular replication in the macrophage-like U937 and J774.1 cells (Supplementary Figure S1), a key factor of *Fth* virulence mainly determined by the type six secretion system (T6SS).

*Survival of* *Fth* *strain A-271 in buffer and in co-culture with amoebae at 25 and 4°C.*

In aquatic habitats the cultiviabilty of *Francisella* is increased at lower temperatures and in the presence of amoebae. We investigated if our WT strain *Fth* A-271 exhibits a similar phenotype. As expected, when *Fth* A-271 was incubated in Ac buffer, CFU was stable over a period of 22 days at 4°C, whereas at 25°C, no further bacterial growth (CFU) could be detected on agar plates after ≥14 days of incubation (Supplementary Figure S2A).

Furthermore, we checked the survival of *Fth* WT strain in Ac buffer, incubated at 4°C or 25°C in the presence of the amoebae *N. gruberi*. At 4°C the CFU values of *Fth* A-271 were stable during the whole experiment, whereas at 25°C no CFU could be detected after 22 to 28 days of incubation (Supplementary Figure S2B). Thus, in the presence of *N. gruberi* the strain was culturable 1 to 2 weeks longer than without amoebae (Supplementary Figures S2A, B). The obtained results indicated that the culturability of our *Fth* WT strain A-271 was increased at lower temperatures and in co-culture with amoebae as previously described for other (virulent) *Francisella* strains (see above).

A number of questions arose, firstly if our *Fth* WT strain would be able to form its own biofilm, then whether biofilm formation might be related to enhanced survival in the environment, and finally if A-271 is able to colonize natural aquatic biofilms.

### Biofilm formed by *in vitro* grown *Fth* A-271 WT strain

#### Biofilm structure

**Figure 1:**
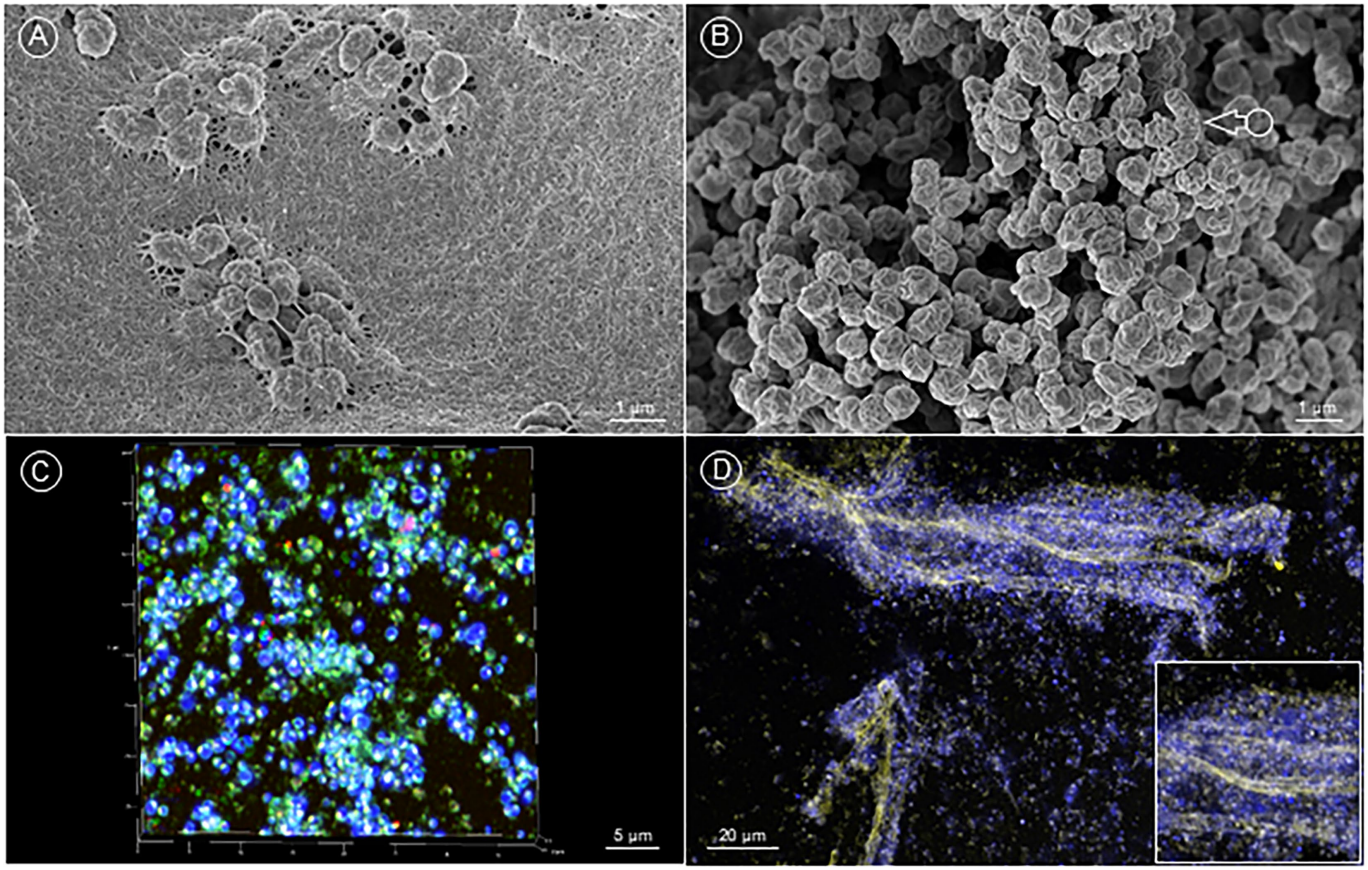
*In vitro* formation of *F. tularensis* biofilm. **(A)** (SEM image) *Fth* grown on collagen coated PE filter on MTK agar plates inoculated with 10^2^ bacteria/filter. Plates were incubated at 37°C with 5% CO_2_. *Fth* formed biofilm with a dense, fibrous extracellular matrix. **(B)** (SEM image) *Fth* biofilm, grown on a Thermanox™ coverslip for 4 weeks showing the characteristic crumpled morphology (arrow). **(C)** (CLSM image) 3D projection of a *Fth* biofilm stained with DAPI (dsDNA), Sypro® Orange (proteins) and Nile Red (neutral lipids). **(D)** *Fth* biofilm with plaited strands of eDNA (yellow) and associated *F. tularensis* bacteria cells (DNA: blue).

When *Fth* strain A-271 was grown *in vitro* for 72 h at 37°C with 5% CO_2_ on agar plates on a polyester filter, SEM analysis revealed the presence of a macrocolony biofilm, in which the bacteria were densely embedded in a luxurious extracellular matrix (Figure 1A). After incubation of *Fth* on coverslips (Thermanox™) in the presence of medium T for four weeks, a similar biofilm structure, although with less biofilm matrix was observed (Figure 1B). We could distinguish micro-and macro-colonies as well as complex 3D biofilm structures. CLSM analysis of this biofilm demonstrated that the matrix was composed of proteins, carbohydrates and lipids (Figure 1C) and also long strands of plaited eDNA, with which *Francisella* bacteria were associated (Figure 1D). Similar biofilm structures were also observed using *Fth* strain LVS or *F*. sp. strain W12-1067 (data not shown).

#### Survival of planktonic or biofilm bacteria of *Fth* A-271 in natural water

**Figure 2:**
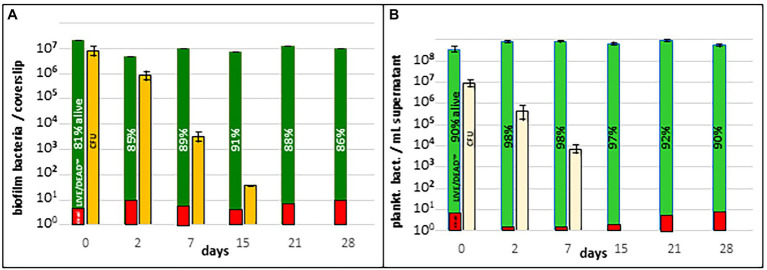
Survival of *Fth* in natural water. **(A)** Survival of *Fth* biofilm in natural water. **(B)** Survival of planktonic *Fth* bacteria in natural water. *Fth* biofilm and respective planktonic cells were incubated in natural water and investigated if the bacteria are still cultivable (CFU) and viable (Live-Dead staining). The results represent the average of two experiments.

*Fth* biofilm (grown on a Thermanox™ coverslip, see above) was then introduced into SNW and incubated at RT for several weeks. On day 0, 2, 7, 15, 21 and 28 the complete biofilm was scraped from the coverslip and planktonic and biofilm bacteria were analyzed for CFU and viability (see methods). Culturability of *Fth* A-271 was measured by CFU on agar plates and viability by Live/Dead staining. Up to 2 days after the release of the biofilm into natural water approx. 85% of the bacteria were alive and the majority was culturable on agar plates (Figure 2A). Whereas the number of living bacteria, as detected with Live/Dead™ staining, did not change significantly over the whole period of the experiment, the number of culturable bacteria (CFU) decreased between week two and three to zero. Similar results were obtained with the planktonic form of *Fth* WT, with the exception that the loss of culturability occurred already between week one to two (Figure 2B). This suggests that planktonic and biofilm forms of *Fth* A-271 survive to a similar amount on the way to a VBNC-like status, even though bacteria in the biofilm status showed a slightly increased culturability over time.

Next, we asked, if the *Fth* WT strain may be able to colonize an existing natural biofilm and survive within this natural habitat.

### *Fth* wild-type is able to colonize a natural multi-species *ex vivo* biofilm (microcosm)

**Figure 3:**
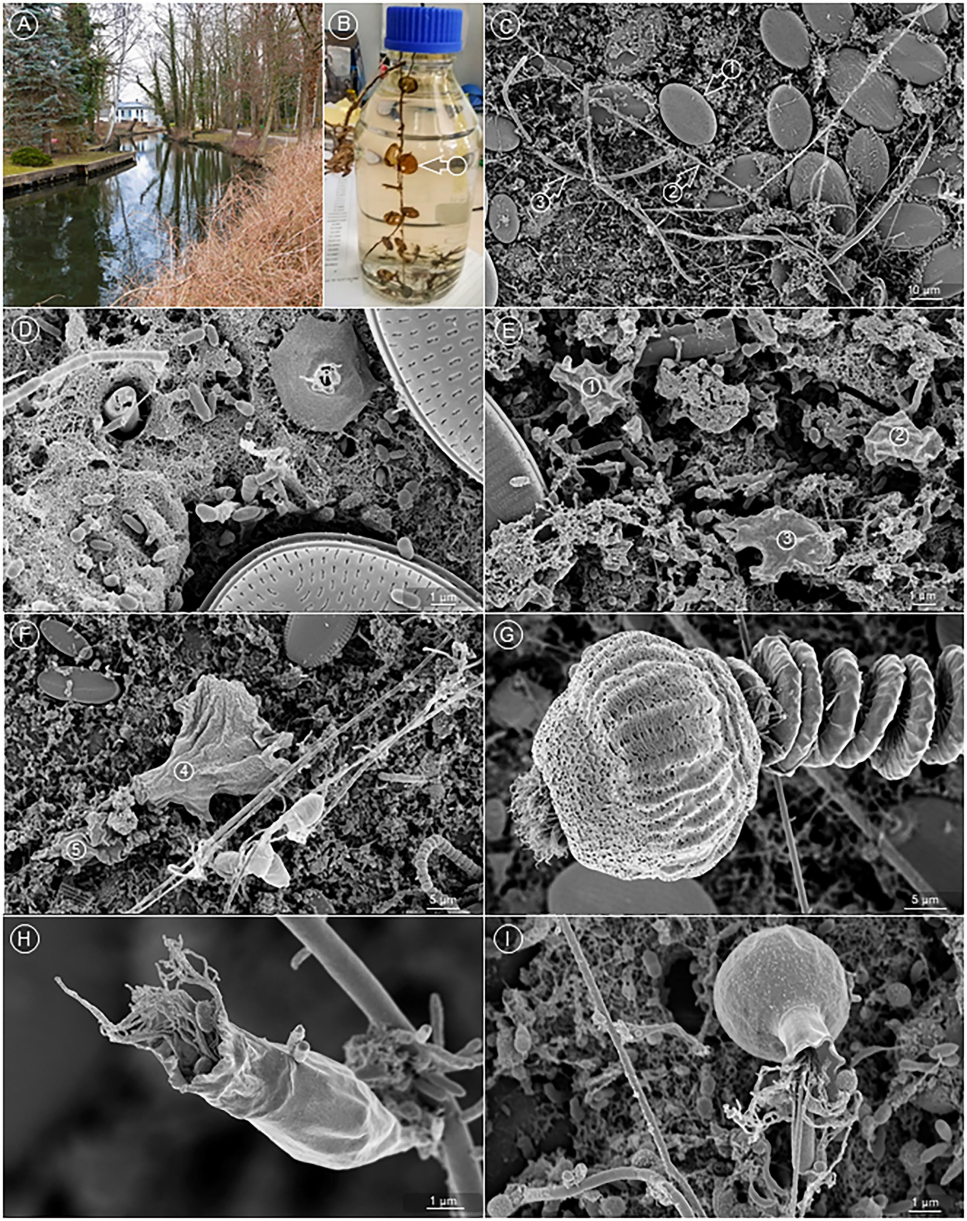
An *ex vivo* biofilm from natural water. **(A)** Sampling location at the Bretter ditch in Brandenburg, Germany. **(B)** Sampled Thermanox™ coverslips (arrow) colonized with aquatic biofilm, transferred into a glass flask for the transport to the laboratory. **(C)** Overview of the biofilm. Readily visible are diatoms (arrow 1), bacteria, wrapped in a long, filamentous sheath (arrow 2), and hyphae of fungi (arrow 3). **(D)** The bottom layer of the biofilm is constituted by numerous bacteria, which are mostly embedded in a fibrous matrix. **(E,F)** Various amoebae were found on top of the bacterial bottom layer (circles 1–5). **(G)** *Vorticella* spp. were frequent constituents of the aquatic biofilm, **(H,I)** as were a great variety of other bacteria eating Ciliates.

In order to test if *Fth* WT is able to colonize natural biofilms under aquatic habitat conditions, we established a natural biofilm on a Thermanox™ coverslip to use this biofilm in *ex vivo* experiments. For 4 weeks, we placed coverslips 20–30 cm beneath the water plane at different seasons in natural aquatic habitats near Berlin (see Materials and methods; Figure 3A). The coverslips were overgrown with biofilms visible with the naked eye (Figure 3B). CLSM imaging of the *ex vivo* biofilm was performed to visually characterize the microbial composition of this biofilm. Microscopy revealed the presence of a bacterial biofilm layer at the bottom (first colonizers), as well as different amoebae and protozoa (Figures 3C–I). For example, we found a wide variety of diatoms (Figures 3C, D) (September), amoebae (Figures 3E, F) (September), and Ciliophorae (Figures 3G–I) (September/November) with bacteria attached to the ciliae (Figures 3H, I) (November). Light microscopy also showed the presence of further protozoa, like Paramecium, Copepods, and further amoebae (data not shown). Altogether the results suggested that an aquatic biofilm was successfully established on the Thermanox™ coverslips during the incubation in the natural aquatic habitat.

**Figure 4:**
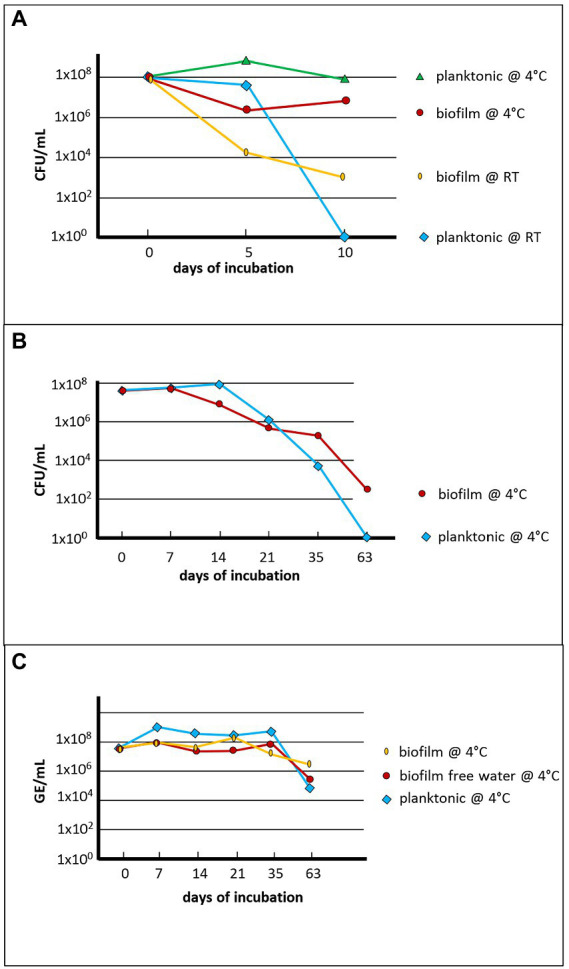
Quantification of *Fth’* survival in natural *ex vivo* biofilm. The number of colony forming units (CFU) of *Fth* and the genome equivalents (GE) were measured over time of the representative experiment conducted in November of the year. **(A)** Comparison of survival of planktonic Fth and Fth in the biofilm at room temperature and at 4°C; **(B)** Long term survival of planktonic Fth and Fth in the biofilm in natural water at 4°C; **(C)** The same experiment as shown in **(B)**, but detection of *Fth* DNA in biofilm, in planktonic supernatant from the biofilm, and in natural water without previous biofilm formation (control). The Thermanox™ coverslips overgrown with or without biofilm in NSW were infected with 4×10^7^ *F. tularensis* on day 0. At several time points as indicated, CFU and GE were determined from the NSW or the supernatant of the coverslips, and of the scraped biofilm (see Material and Methods).

Then these coverslips were infected with 10^7–8^ GFP-labelled *Fth* A-271 bacteria and investigated the amount of culturable *Fth* cells in co-culture with this biofilm (in autoclaved natural water) for up to 63 days. Results are given in Figure 4. As in water, the time span of culturability of the bacteria was decreased at RT compared to 4°C (Figure 4A). The culturability of *Fth* at 4°C in the presence of the biofilm was slightly increased compared to *Fth* in water (Figure 4B) and the detectable genomic units were only reduced two to three log_10_ levels over the whole period of incubation (Figure 4B).

**Figure 5:**
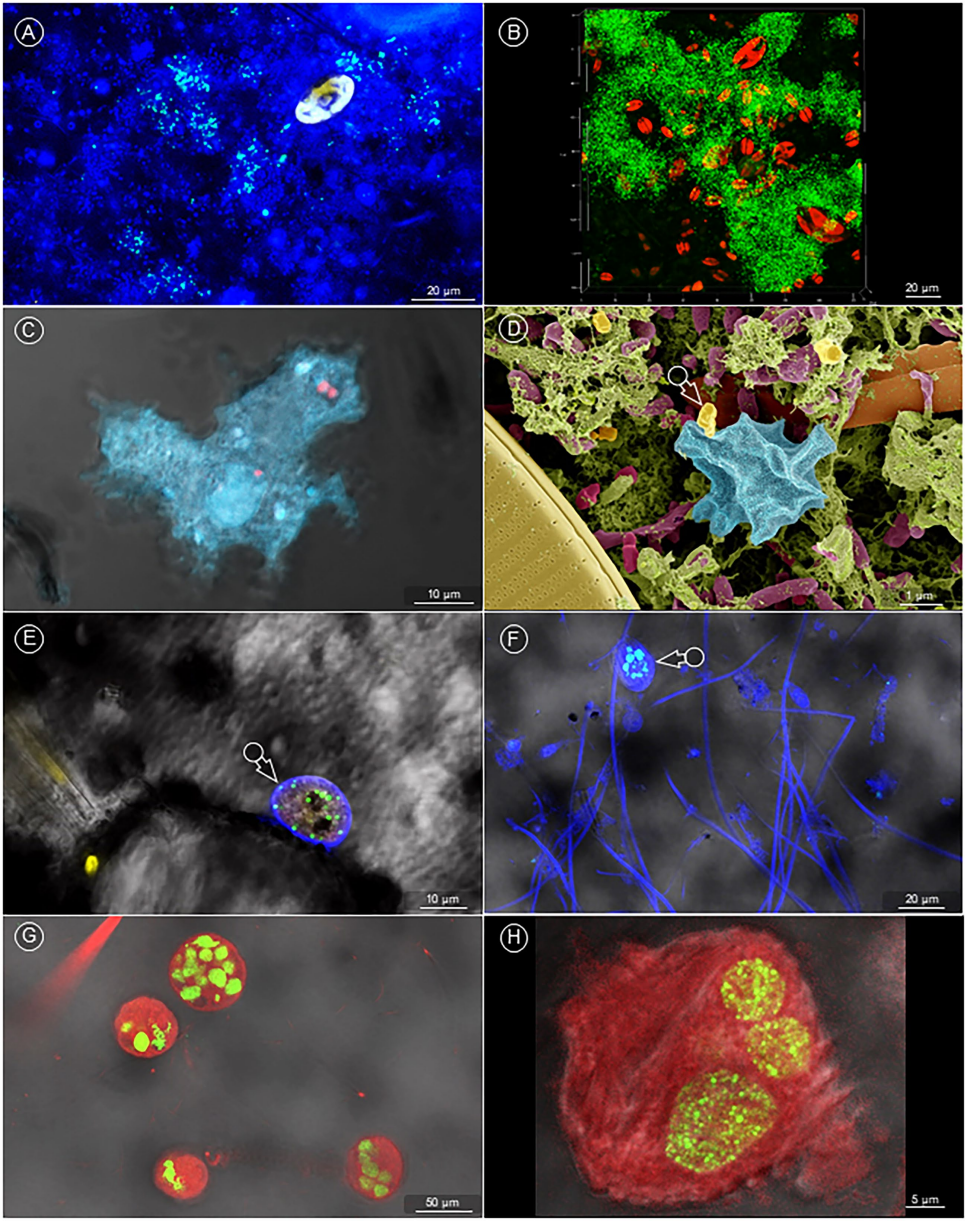
Visualization of *Fth’* survival in natural *ex vivo* biofilm. **(A)** (CLSM image) GFP expressing *Fth* formed microcolonies (blue-greenish signal) within the natural biofilm (DAPI, dark blue) – diatom: yellowish autofluorescence). **(B)** (CLSM image) *Fth* formed a comprehensive, 3D biofilm (green) within the natural biofilm – diatoms: red autofluorescence. **(C)** (CLSM image) In the natural biofilm, *Fth* bacteria (red) were found ingested by amoebae (bright blue). **(D)** (SEM image) A bacterium, with the characteristic shape of *Fth* (arrow, compare with Figure 2B), is about to be ingested by an amoeba (bright blue). This scene enfolds between matrix (green) embedded bacteria (dark red) and various diatoms (ocker and orange). **(E,F)** (CLSM image) GFP expressing *Fth* bacteria within different types of ciliates (arrows). **(G,H)** Many *Vorticella* (red autofluorescence) in the natural biofilm showed accumulated *Fth*, which formed dense clusters (green).

When the same experiment – *ex vivo* biofilm, co-incubated with *Fth* A-271 at 4°C – was imaged with the CLSM, *Fth* bacteria after 10 days of incubation were most commonly visible as single bacteria, micro- and macrocolonies in the bacterial bottom layer of the *ex vivo* biofilm (Figure 5A, November-biofilm). On few occasions, *Fth* had even formed their own comprehensive, three-dimensional biofilm structure (Figure 5B, July-biofilm, 10^th^ day of incubation). More frequently, *Fth* bacteria were found in amoebae (Figure 5C, November-biofilm, 5th day of incubation), or (presumably) about to be taken up by one (Figures 5D, E) (September, 5th day of incubation). Regularly, *Fth* was also spotted in a wide variety of ciliates (Figures 5E, F) (November, 5th day of incubation), in particular in *Vorticella* spp. (Figures 5G, H) (November, 10th day of incubation) as single bacteria or clustered in vacuoles (Figures 5F–H).

Taken all together, the results clearly demonstrated that *Fth* is able to successfully colonize different niches of an existing natural biofilm by forming macro-colonies, but also complex biofilm structures of their own.

## Discussion

Many studies about *Francisella* were performed with the *Fth* strain LVS, which is a virulence attenuated strain and its laboratory handling is much easier. Although strain LVS is used as a surrogate for *F. tularensis*, this strain is not fully virulent and may not behave as a *Fth* WT strain (Rohmer et al., 2006; Biot et al., 2020; Mlynek et al., 2021). Thus, we used the *Fth* WT strain A-271, isolated from a carcass of a beaver deceased from tularemia, in our experiments (Schulze et al., 2016; Sundell et al., 2020). Here we demonstrated that this strain is a virulent *Fth* WT strain and able to replicate in macrophage-like cell lines. In addition, *in silico* analysis of the genome of this strain confirms the presence of all common virulence factors of *Fth* (Sundell et al., 2020). Furthermore, using this isolate (*Fth* A-271), we corroborate earlier findings showing that the culturability of *Francisella* over time in water is improved at lower (4°C) temperatures rather than at higher ones (RT, 22.5°C). Furthermore, using the amoeba *Naegleria gruberi* we here can confirm prior publications, demonstrating that *Francisella* is not able to multiply in amoebae (*Acanthamoeba castellanii*, *A. polyphaga*, *Vermamoeba vermiformis*) (Buse et al., 2017; Hennebique et al., 2021). However, lower temperatures and the presence of amoeba increased the survival and the long-term culturability of the bacterium (Abd et al., 2003; El-Etr et al., 2009; Duodu and Colquhoun, 2010; Verhoeven et al., 2010; Gilbert and Rose, 2012; Ozanic et al., 2016; Buse et al., 2017; Golovliov et al., 2021; Hennebique et al., 2021).

Therefore, we chose *Fth* A-271 WT strain to investigate its ability to form biofilms and to colonize and survive in a natural aquatic multi-species biofilm.

### Biofilm formation

Strain *Fth* A-271 grown on agar plates or in medium T on Thermanox™ coverslips was able to form micro- and macro-colonies, as well as a 3D biofilm structure with a large amount of matrix material, containing strands of eDNA, carbohydrates, proteins and lipids. These results demonstrate the ability of this strain to form its own biofilm (Figures 1A, C). Investigating the survival of bacteria in natural water, the experiments demonstrated that the survival of bacteria in biofilms and planktonic bacteria seem to be similar, but the time-span of culturability of biofilm bacteria was increased compared to their corresponding planktonic form (Figure 2). This indicates that bacteria within the biofilm stay culturable for a longer time as also shown for *F. novicida* and *F. philomiragia* or for *Francisella* in co-culture with amoebae (Verhoeven et al., 2010; Ozanic et al., 2016; Buse et al., 2017; Siebert et al., 2020; Hennebique et al., 2021; Mlynek et al., 2021). The results suggest that biofilm formation may also enhance *Francisella* persistence within an aquatic habitat.

### Colonization of a natural aquatic biofilm (microcosm)

We mentioned above that *Fth* WT strain A-271 in our experiment slowly lost its culturability and that they formed cells in a VBNC-like state (Figure 4). VBNC has been described for *Francisella*, but to our knowledge, the ability to resuscitate these forms has not been published for *Francisella* so far (Forsman et al., 2000; Thelaus et al., 2009; Duodu and Colquhoun, 2010; Backman et al., 2015). For *Legionella pneumophila* in contrast, it has been shown that resuscitation of bacteria in the VBNC status is possible in amoebae (Steinert et al., 1997; Epalle et al., 2015). However, *Francisella* is able to survive and persist over long time periods in natural aquatic environments in a temperature-dependent manner (Parker et al., 1951; Forsman et al., 2000; Sinclair et al., 2008; Berrada and Telford Iii, 2011; Broman et al., 2011; Gilbert and Rose, 2012; Golovliov et al., 2021).

Furthermore, it was demonstrated that co-cultures of *Francisella* and amoebae increase the culturability but not the virulence of *Francisella*, as *Francisella* is not able to multiply within amoebae (Abd et al., 2003; El-Etr et al., 2009; Verhoeven et al., 2010; Ozanic et al., 2016; Buse et al., 2017; Hennebique et al., 2021). In addition, microcosm experiments revealed that *Francisella* did not survive the grazing of the ciliate *Tetrahymena pyriformis*, while a nanoflagellate was found to favor *Fth* survival (Thelaus et al., 2009). In addition, *F. noatunensis* survival was shown to be higher in sterile than in nonsterile microcosms (Duodu and Colquhoun, 2010) and survival seems to be dependent on the number and species of bacterial grazing protozoa (Mironchuk Iu and Mazepa, 2002; Thelaus et al., 2009; Duodu and Colquhoun, 2010); and own unpublished results). Thus, the survival of *Francisella* in the aquatic habitat is influenced by a high amount of different biotic and abiotic factors and therefore, we investigated the ability of *Fth* to colonize a multi-species biofilm.

It has been demonstrated that multi-species biofilms are more resistant against stress compared to single-species biofilms (Joshi et al., 2021; Wicaksono et al., 2022), but so far this has not been shown for *Francisella*. As mentioned above, the strain *Fth* A-271 was isolated from a dead beaver living in an aquatic habitat. Thus we analyzed the ability of this strain to survive and to colonize a natural aquatic multi-species biofilm, in a microcosm-like experiment. The coverslips were incubated for 4 weeks in a natural aquatic habitat at different seasons. We characterized these biofilms as a multi-species biofilm, exhibiting diatoms, *Vorticellidae*, *Rotifera*, as well as different amoebae and bacteria. Interestingly, these biofilms could be colonized by our *Fth* WT strain and we could observe micro- and macro-colonies, as well as mature biofilm-like structures of *Fth* within the natural biofilm. Furthermore, as demonstrated for the co-culture with amoebae, the presence of the natural biofilm increased the culturability of *Fth*; and the effect was found also to be temperature-dependent (4°C > RT). Recently, research started to investigate general interspecies interactions (e.g., an biofilm adapted metabolism) within multi-species biofilms (Joshi et al., 2021; Wicaksono et al., 2022) which may be also important for the persistence of *Francisella* in the environment.

In this study, we demonstrated that *Fth* strains are able to successfully colonize natural-like aquatic multi-species biofilms, a behavior which seems to be important for their observed long-term survival within aquatic habitats.

To our knowledge, we are the first to investigate if a *Fth* WT strain is able to colonize and survive within a natural aquatic multi-species biofilm. We can demonstrate that amoebae, biofilm formation and low temperatures increase the culturability (survival) of *Fth* in the aquatic environment and that *Fth* is, indeed, able to successfully colonize a multi-species biofilm. This may have impact on the long-term survival of *Francisella* in aquatic habitats and should be further investigated.

## Data availability statement

The raw data supporting the conclusions of this article will be made available by the authors, without undue reservation.

## Author contributions

CS, KR, and BM performed the experiments. RG, CS, and KH supervised the work. KH, RG, and CS drafted the manuscript. All authors contributed to the revision of the manuscript and read and approved the submitted version.

## Conflict of interest

The authors declare that the research was conducted in the absence of any commercial or financial relationships that could be construed as a potential conflict of interest.

## Publisher’s note

All claims expressed in this article are solely those of the authors and do not necessarily represent those of their affiliated organizations, or those of the publisher, the editors and the reviewers. Any product that may be evaluated in this article, or claim that may be made by its manufacturer, is not guaranteed or endorsed by the publisher.

## Footnotes

^1^http://takarabio.com/

^2^https://imagej.nih.gov/ij/
