# Supplementary material for: Francisella tularensis subsp. holarctica wild-type is able to colonize natural aquatic ex vivo biofilms
Source: Front Microbiol. 2023 Feb 13;14:1113412. doi: 10.3389/fmicb.2023.1113412 (PMC9969146; doi:10.3389/fmicb.2023.1113412)
Supplement: Supplementary file 1 [file Presentation_1.PPTX]

## Slide 1
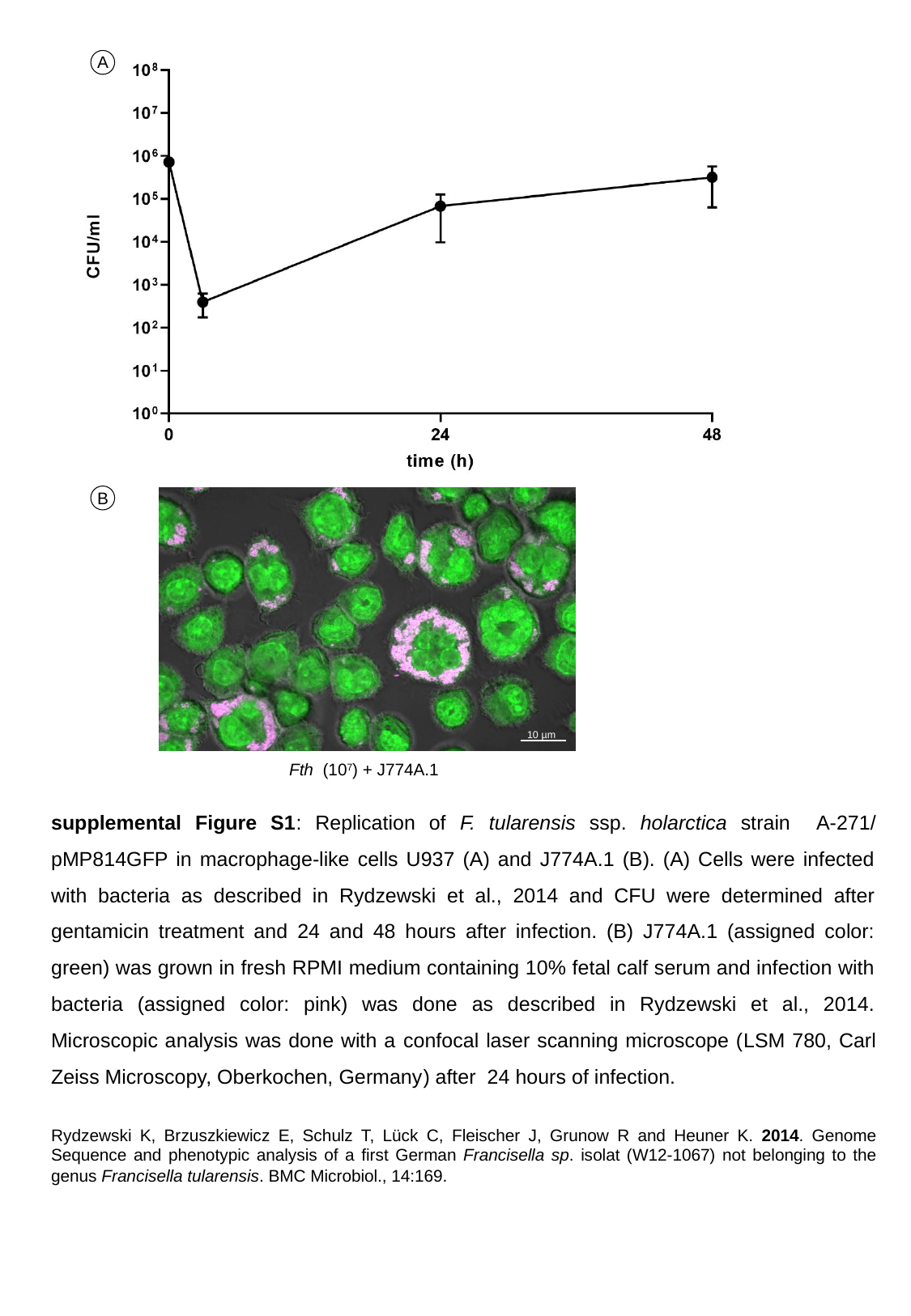

A
3
B
10 µm
10 µm
Fth (107) + J774A.1
supplemental Figure S1: Replication of F. tularensis ssp. holarctica strain A-271/ pMP814GFP in macrophage-like cells U937 (A) and J774A.1 (B). (A) Cells were infected with bacteria as described in Rydzewski et al., 2014 and CFU were determined after gentamicin treatment and 24 and 48 hours after infection. (B) J774A.1 (assigned color: green) was grown in fresh RPMI medium containing 10% fetal calf serum and infection with bacteria (assigned color: pink) was done as described in Rydzewski et al., 2014. Microscopic analysis was done with a confocal laser scanning microscope (LSM 780, Carl Zeiss Microscopy, Oberkochen, Germany) after 24 hours of infection.
Rydzewski K, Brzuszkiewicz E, Schulz T, Lück C, Fleischer J, Grunow R and Heuner K. 2014. Genome Sequence and phenotypic analysis of a first German Francisella sp. isolat (W12-1067) not belonging to the genus Francisella tularensis. BMC Microbiol., 14:169.
