# Supplementary material for: Francisella tularensis subsp. holarctica wild-type is able to colonize natural aquatic ex vivo biofilms
Source: Front Microbiol. 2023 Feb 13;14:1113412. doi: 10.3389/fmicb.2023.1113412 (PMC9969146; doi:10.3389/fmicb.2023.1113412)
Supplement: Supplementary file 2 [file Presentation_2.PPTX]

## Slide 1
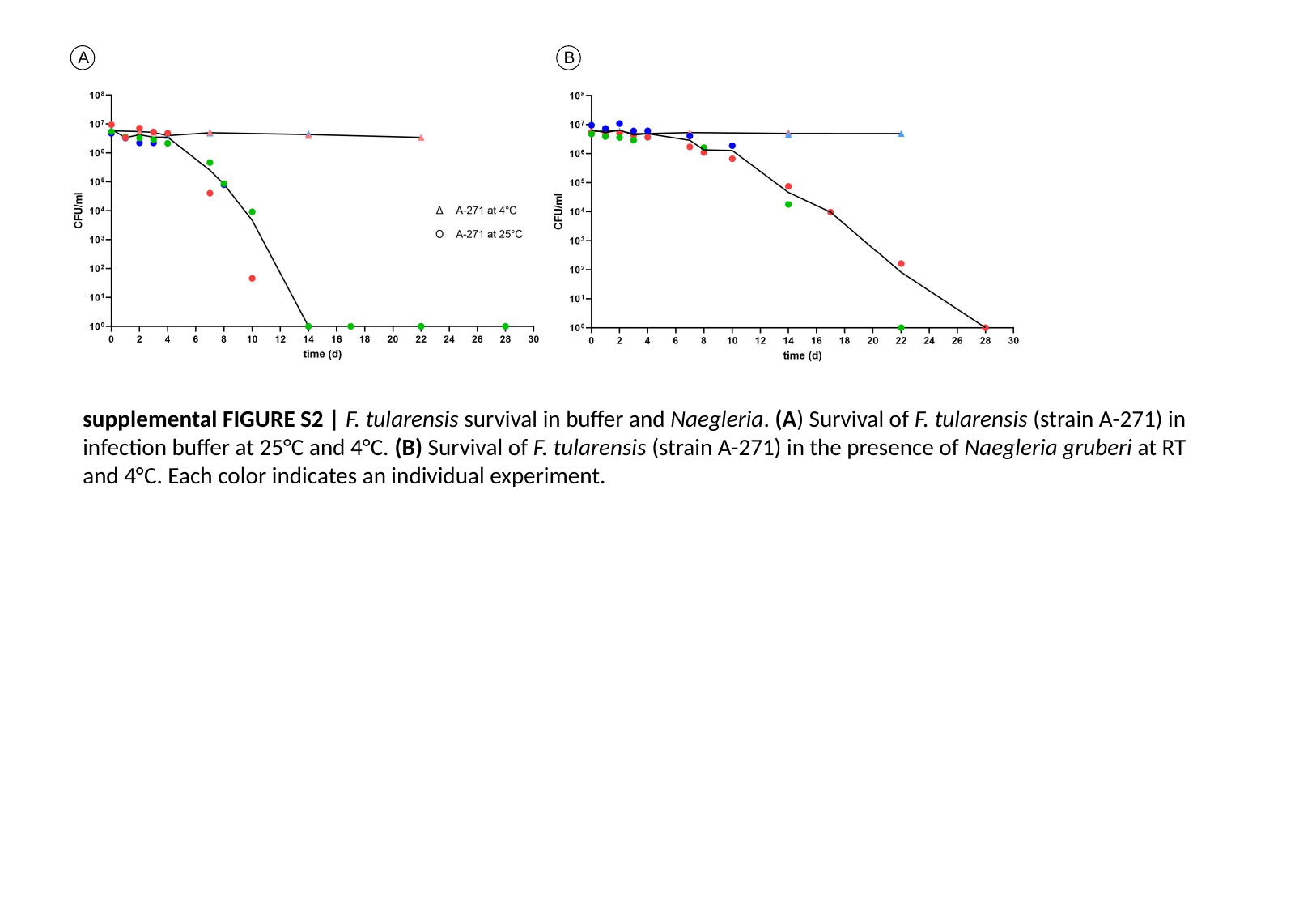

A
B
supplemental FIGURE S2 | F. tularensis survival in buffer and Naegleria. (A) Survival of F. tularensis (strain A-271) in infection buffer at 25°C and 4°C. (B) Survival of F. tularensis (strain A-271) in the presence of Naegleria gruberi at RT and 4°C. Each color indicates an individual experiment.
