# Supplementary figures and images for: Francisella tularensis subsp. holarctica wild-type is able to colonize natural aquatic ex vivo biofilms
Source: Front Microbiol. 2023 Feb 13;14:1113412. doi: 10.3389/fmicb.2023.1113412 (PMC9969146; doi:10.3389/fmicb.2023.1113412)

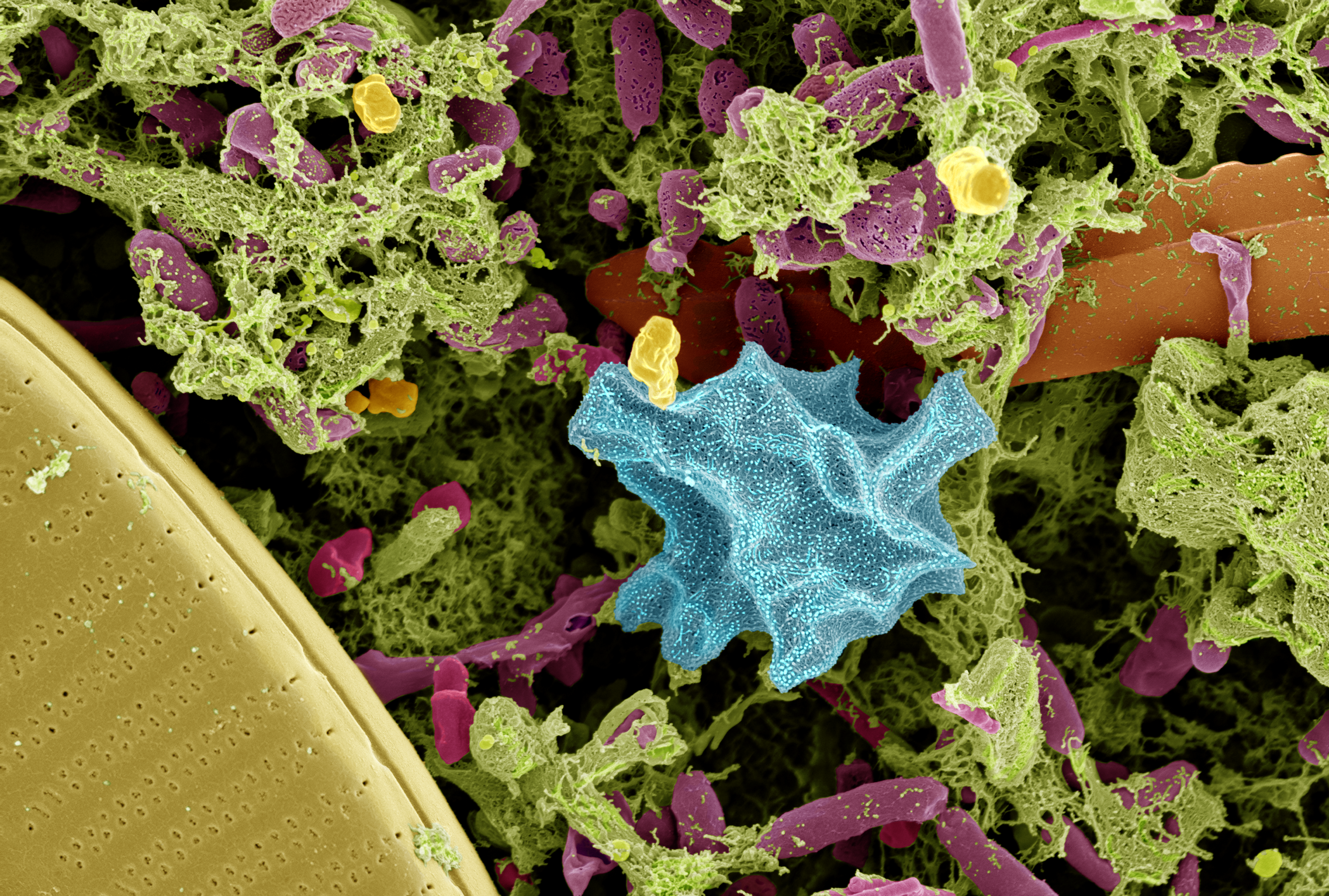

Supplement: Supplementary file 3 [file Image_1.TIF]
